# Silicone pneumonitis after gluteal filler: a case report and literature review

**DOI:** 10.1002/rcr2.538

**Published:** 2020-02-18

**Authors:** Boon Hau Ng, Wan Rahiza Wan Mat, Nik Nuratiqah Nik Abeed, Mohamed Faisal Abdul Hamid, Andrea Ban Yu‐Lin, Chun Ian Soo

**Affiliations:** ^1^ Pulmonology Unit, Department of Internal Medicine, Faculty of Medicine Universiti Kebangsaan Malaysia Medical Centre Kuala Lumpur Malaysia; ^2^ Department of Anesthesiology and Intensive Care Unit, Faculty of Medicine Universiti Kebangsaan Malaysia Medical Centre Kuala Lumpur Malaysia

**Keywords:** Acute respiratory distress syndrome, gluteal filler, silicone embolization syndrome, silicone pneumonitis

## Abstract

Liquid silicone (polydimethylsiloxane) is an inert material that is commonly used for cosmetic purpose. Silicone embolization syndrome (SES) can rapidly progress to pneumonitis as a consequence of the injection of nonmedical‐grade liquid silicone. We describe a case of severe silicone pneumonitis complicated with acute respiratory distress syndrome and bilateral pneumothorax secondary to silicone gluteal augmentation. In this case report, we aim to discuss our experience and approach in managing an uncommon case of SES.

## Graphical abstract

We describe a case of severe silicone pneumonitis complicated with acute respiratory distress syndrome and bilateral pneumothorax secondary to silicon gluteal augmentation. In this case report, we aim to discuss our experience and approach in managing an uncommon case of silicone embolization syndrome.

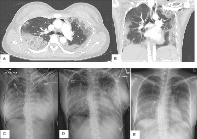

## Introduction

For decades, Liquid injectable silicone has been used for correction of contour defect or soft‐tissue augmentation. Medical‐grade liquid silicone (polydimethylsiloxane) becomes the preferred inert material for cosmetic purpose due to its durability, a lack of immunogenicity, and thermal stability; but later found to be associated with silicone embolism syndrome (SES). Liquid injectable silicone induced embolism has been reported by several studies as a cause of acute pneumonitis with alveolar haemorrhage 1, 2, 3.

## Case Report

A previously healthy 30‐year‐old woman presented with three days history of cough with dyspnoea and fever. She had a history of breast augmentation with silicone implant two years ago and had received bilateral gluteal silicone injections from an unlicensed provider one week before the current presentation. The injected volume was approximately 500 mL for each gluteal.

The physical examination was tachycardia, tachypnoea with the respiratory rate of 28 breaths per minute and a temperature of 38°C. Results of the arterial blood gas performed while the patient was breathing room air were as follows: pH, 7.39; PaCO_2_, 40 mmHg; PaO_2_, 56 mmHg; and peripheral oxygen saturation, 90%. Lungs examination revealed bilateral lower zone crepitations. Other blood investigations which included a complete blood count, comprehensive metabolic panel, and lactate were unremarkable. She was initiated on broad‐spectrum antibiotics, oseltamivir, intravenous hydrocortisone 50 mg every 8 h, and 12 L/min (FiO2: 60%) of high‐flow oxygen supplement. However, she developed right‐sided pneumothorax and worsened respiratory failure 12 h later.

**Figure 1:**
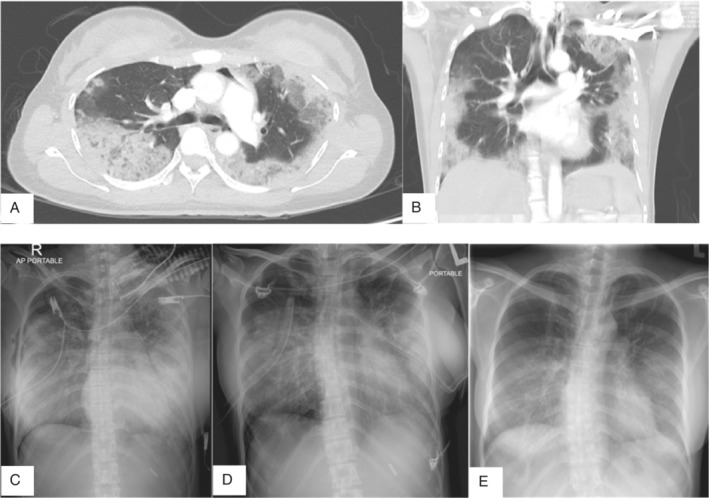
A computed tomography scan of the chest (A and B) shows bilateral, diffusely distributed ground‐glass opacities with superimposed dependent areas of consolidation. Chest X‐ray (C) shows diffuse alveolar opacities of both lung fields. (D) Chest X‐ray with bilateral alveolar opacities and pneumothorax with chest tube in‐situ. (E) Chest X‐ray improvement of bilateral alveolar opacities at 6 weeks of outpatient follow‐up.

**Figure 2:**
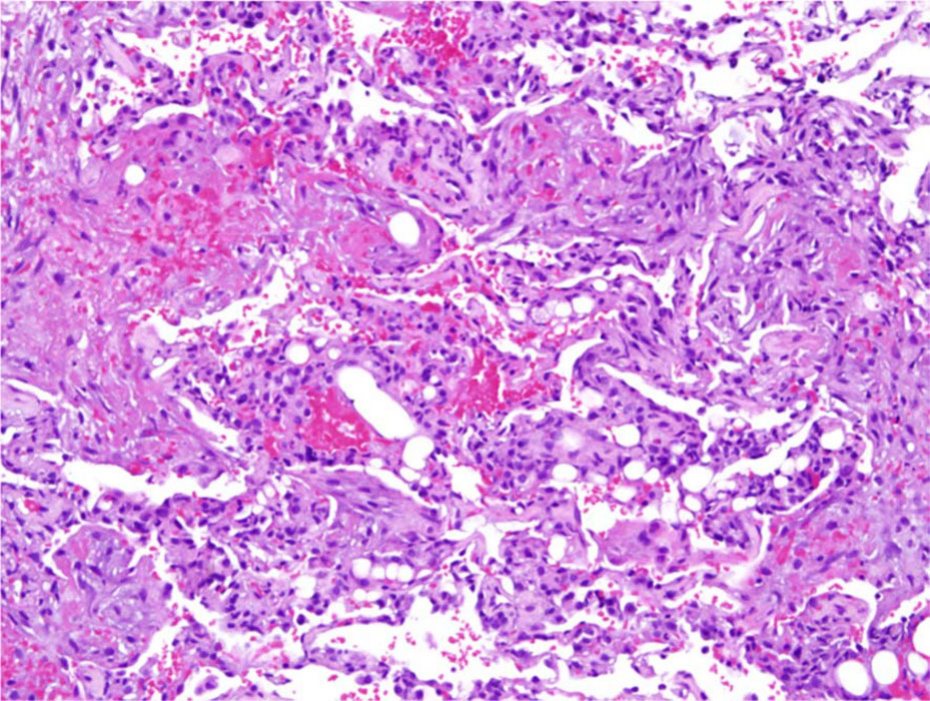
Trans‐bronchial lung biopsy shows lung parenchyma with intra‐alveolar haemorrhage, macrophages, and non‐refractile vacuole‐like structures.

On chest radiograph, diffuse alveolar opacities in both lung fields were observed. A computed tomographic scan of her thorax demonstrated diffuse bilateral ground‐glass infiltrates (Fig. 1). Fiberoptic bronchoscopy was performed, which revealed normal lung segments. Trans‐bronchial lung biopsy showed non‐refractile lipoid vacuoles, consistent with silicone pneumonitis (Fig. 2). Magnetic resonance imaging of the breast confirmed no intracapsular and extracapsular rupture of the bilateral breast prosthesis. Her bronchial wash was positive for Coronavirus NL63 RNA PCR.

She received lung protective ventilation for acute respiratory distress syndrome (i.e. low tidal volumes and high positive end‐expiratory pressure (PEEP)) for continued hypoxia. Unfortunately, two weeks later, she again developed another spontaneous pneumothorax on the left lung and required chest tube drainage. Over four weeks in the intensive care unit, we manage to wean down the ventilatory support. She had an excellent clinical response and discharged with tapering prednisolone over two weeks. At six weeks of outpatient follow up, she was symptom‐free, and her chest X‐ray revealed residual bilateral reticulations.

## Discussion

The respiratory consequences that associated with silicone injection include acute pneumonitis 4, acute respiratory distress syndrome, alveolar haemorrhages, and pulmonary embolism 5. The respiratory symptoms generally present within 72 h after injection of higher dose silicone, and a delayed reaction can be seen up to a year and a half after injection 2.

Pulmonary SES results from silicone embolizing to the lungs from an inadvertent intravenous injection or local tissue destruction. The neutrophils ingested the embolization of the silicone material into the lung via the haematogenous or lymphatic and alveolar macrophages induces pneumonitis by local cell‐mediated inflammation and release of free radicals and proteolytic enzymes 3. The release of silicone emboli also leads to occlusion of the microvasculature and trigger the inflammatory response, resulting in pulmonary oedema and haemorrhage.

Diagnosis of the silicone pneumonitis is based on the clinical history of silicone implant or injection, the radiological pattern of subpleural infiltrates and peripherally distributed ground‐glass opacities (GGO) 6, 7, and tissues biopsy with histopathological features of alveolar haemorrhage 7 and non‐refractile vacuole‐like structure within the alveoli 1. Presence of alveolar macrophages with intracytoplasmic silicone inclusions in bronchoalveolar lavage sample is useful in facilitating a diagnosis 3.

Survival is likely, and treatment involves ventilation if necessary and steroids with uncertain utility. The rate of mortality is directly associated with the large volumes of silicone and high‐pressure injection 2.

There is no consensus on the treatment of the silicone induced pneumonitis, but case report series show a favourable response with the use of the steroid (Table 1) to reduce the airways inflammation 1, 8, 9. General measures include nutrition and ventilation support. Extracorporeal membrane oxygenation (ECMO) may be considered in cases of severe acute respiratory distress syndrome (ARDS) deteriorating on mechanical ventilation. Surgical extraction of the silicone from subcutaneous tissues have had poor outcome due to risk of adverse systemic effects and technically complicated surgery 10.

**Table 1:** Reported cases of pulmonary silicone embolism syndrome.

Author	Year	Type of silicone application	Injection site	Symptoms onset after implant	Radiology findings	Complications	Treatment	Survival
Carolyn et al. 11	2019	Injection	Gluteal	2 days	Diffuse GGO	Pneumonitis, ARDS	MTP 125 mg every six hours, ECMO	Yes
Ahad et al. 17	2019	Implant	Breast	14 years	Alveolar infiltrates	Pneumonitis	Corticosteroids, ECMO, antibiotics	No
Srilekha et al. 12	2018	Injection	—	15–20 years	Diffuse GGO	Pneumonitis, pneumothorax	Corticosteroids	No
Elizabeth et al. 13	2018	Implant	Breast	16 years	Hilar & mediastinal LN, pulmonary nodules	Pulmonary nodules	Prednisone 40 mg/day tapering down over 6 months	Yes
		Implant	Breast	2 years	Diffuse micro‐nodules with GGO	Pneumonitis	Implant removal, prednisone 40 mg/day	Yes
		Implant	Breast	12 years	Diffuse GGO and reticular opacities	Organising pneumonia	Prednisone 40 mg/day with tapering down over 6 months and azathioprine as steroid sparing agent	Yes
Arthur et al. 18	2018	Implant	Breast	6 months	Alveolar infiltrates, diffuse GGO	Pneumonitis, PH	Implant removal, prednisolone 40 mg/day, ECMO	Yes
Rafael et al. 19	2017	Implant	Gluteal	4 months	—	Granulomatous inguinal LN	Capsulotomy	Yes
María et al. 20	2016	Implant	Breast	10 years	Consolidation	Pneumonitis	Implant removal, corticosteroids	Yes
Kirill et al. 21	2016	Injection	Gluteal	2 months	Diffuse GGO	Pneumonitis	Supportive	Yes
		Injection	Gluteal	8 months	Diffuse GGO, mediastinal & hilar LN	Pneumonitis	Supportive	Yes
Ayush et al. 22	2016	Implant	Breast	18 years	Diffuse GGO	Pneumonitis	Implant removal, corticosteroids	Yes
Erin et al. 23	2015	Injection	Gluteal	5 months	Alveolar infiltrates	Gluteal abscess, ARDS	Incision and drainage, antibiotics	Yes
Alex et al. 24	2013	Injection	Gluteal	2 days	Diffuse GGO	Pneumonitis, PH	MTP 125 mg every six hours	Yes
Dercio et al. 25	2012	Injection	Gluteal	2 week	Alveolar infiltrates	Pneumonitis	Corticosteroids	Yes
Denyo et al. 26	2012	Injection		36–48 h	Alveolar infiltrates	Pneumonitis, PH	—	‐
		Injection		36–48 h	Alveolar infiltrates	Pneumonitis, PH	—	‐
Priya et al. 27	2011	Injection	Gluteal	6 h	Diffuse GGO	Pneumonitis, PH	Supportive	Yes
Sophie et al. 28	2010	Injection	Gluteal, hip	1 day	Noncalcified pulmonary nodules, GGO	Pneumonitis	MTP	Yes
Rupen et al. 29	2008	Injection	Thigh	3 days	Alveolar infiltrates	Pneumonitis	MTP	Yes
Richard et al. 30	2008	Injection	Gluteal, thigh, face	4 h	Alveolar infiltrates	Organising pneumonia, ARDS	MTP	No
Rafael et al. 31	2007	Injection	Breast	40 h	Alveolar infiltrates	Pneumonitis, ARDS	Supportive	No
Grigoriy et al. 32	2006	Injection	Gluteal	12 days	Alveolar infiltrates	Pneumonitis, PH	MTP 250 mg every six hours	Yes
Samuel et al. 33	2006	Injection	Gluteal	—	Subpleural GGO and consolidation	Pneumonitis, PH	Supportive	‐
Alex et al. 34	2004	Injection	Breast	1 week	Alveolar infiltrates	Pneumonitis, PH	Corticosteroids	Yes
Cheol et al. 35	2003	Injection	Vaginal colpoplasty	2 days	Interstitial infiltrates, air‐space consolidation	Pneumonitis	Corticosteroids	Yes
Jean et al. 3	1983	Injection	Trochanter	3 days	—	Pneumonitis	Supportive	Yes
		Injection	Trochanter	2 days	—	Pneumonitis	Supportive	Yes
		Injection	Trochanter	1 day	Interstitial infiltrates, air‐space consolidation	Pneumonitis	Supportive	Yes

ARDS, acute respiratory distress syndrome; ECMO, extracorporeal membrane oxygenation; GGO, ground glass opacities; LN, lymphadenopathy; MTP, methylprednisolone; PH, pulmonary haemorrhages.

Carolyn et al. reported a case of acute pneumonitis after silicone injection for gluteal augmentation. The patients presented with haemoptysis, shortness of breath, and acute respiratory failure two days after the silicone injections 11. Her computed tomography (CT) thorax showed predominantly basilar and peripheral GGO and pulmonary nodules bilaterally. She required ECMO and her condition improved with intravenous methylprednisolone 125 mg every 6 h.

Srilekha Sridhara et al. reported a rare case of silicone pneumonitis with pneumothorax occurred even after 15–20 years of silicone injection 12. Bronchoscopic lung biopsy demonstrated alveolar interstitium with numerous lipoid vacuoles, compatible with silicone deposition. The patient succumbed despite treated with intravenous corticosteroids, broad‐spectrum antibiotics, and mechanical ventilation.

Elizabeth et al. reported three cases of breast implants with the onset of symptoms ranges 2–16 years 13. The CT features of these patients demonstrated diffuse GGO. All three patients responded to prednisolone 40 mg/day.

Our case demonstrates the features of SES after the gluteal silicone filler. No specific site for vascular infiltration was identified in our case; however, many injection marks were observed on the buttocks. MRI breast confirmed that there is no leak from the silicone breast implants and no residual collection in the gluteal region. Thus, the decision not to remove the breast implant and not for surgical exploration of the possibilities of remaining gluteal silicone collection was made after a multidisciplinary meeting comprises of pulmonologist, plastic and reconstructive surgeon, and anaesthetic team. A trans‐bronchial lung biopsy was deemed to be a less invasive approach to obtain lung biopsies in order to avoid the risk of general anaesthesia due to poor pulmonary reserve.

The detection of HCoV‐NL63 in our patient is incidental. This is consistent with the literature that HCoV‐NL63 can be frequently found in asymptomatic individuals 14. Although, HCoV‐NL63 can infects both the upper and lower respiratory tract, it generally associated with mild symptoms, such as fever, cough, pharyngitis, and rhinitis 15. In rare cases, pneumonia can occur. In total contrast to silicon pneumonitis, lung biopsy histopathological features of HCoV‐NL63 pneumonia are chronic pulmonary inflammation, severe alveolar damage, intra‐alveolar hyaline membranes, and interstitial oedema 16. The result of the lung biopsy of our patient was consistent with the diagnosis of silicone pneumonitis.

Learning points for our case includes:

1. Corticosteroids are potentially beneficial in reduces the inflammation of the airways that leading pneumonitis with acute respiratory distress syndrome but does not have any mortality benefits 3, 14.
2. Lung‐protective ventilation strategies improve survival.
3. Vigilance in the occurrence of pneumothorax in cases of SES especially if complicated with ARDS and requiring mechanical ventilation. Risk of pneumothorax in SES and will usually resolve after chest drain intervention.
4. Trans‐bronchial lung biopsy is useful in assisting the diagnosis.
5. Patients should be advised that there is a risk that silicone injections can be associated with serious pulmonary complications.

The injection of silicone for cosmetic purpose is debatable due to the sequelae of the pneumonitis and the potential for pulmonary toxicity in asymptomatic patients who receive silicone injections. Bronchoalveolar lavage and trans‐bronchial lung biopsy can help to confirm the diagnosis. This case report serves to highlight an emergent danger associated with illicit silicone use for cosmetic purposes and clinicians should be aware of the potential complications.

### Disclosure statement

Appropriate written informed consent was obtained for publication of this case report and accompanying images.
